# Clinical and Radiological Evaluation of Warfarin-Associated Intracerebral Hemorrhage (ICH) in Patients With Mechanical Prosthetic Heart Valves: A Retrospective Observational Study

**DOI:** 10.7759/cureus.109377

**Published:** 2026-05-21

**Authors:** Abhishek Kumar, Aditya Bidwaikar, Sheetal Das Panika, Nikhil Raj

**Affiliations:** 1 Cardiology, Government Superspeciality Hospital, Chhattisgarh Institute of Medical Sciences, Bilaspur, IND; 2 Anesthesiology, Government Superspeciality Hospital, Chhattisgarh Institute of Medical Sciences, Bilaspur, IND; 3 Anesthesiology, Chhattisgarh Institute of Medical Sciences, Bilaspur, IND; 4 Neurology, Government Medical College, Kozhikode, IND

**Keywords:** computed tomography, functional recovery, intracerebral hemorrhage, mechanical prosthetic heart valve, mortality, warfarin

## Abstract

Introduction: Intracerebral hemorrhage (ICH) is a serious complication of oral warfarin in patients with mechanical heart valves, leading to high mortality and disability. This study aimed to evaluate the clinical and radiological features and the variables associated with patient outcomes in such cases, particularly in resource-limited settings.

Methods: This retrospective observational study included 125 patients on warfarin with mechanical heart valves who developed spontaneous ICH and presented within 24 hours of symptom onset. Hematoma volume was calculated using the ABC/2 method. Outcomes were assessed at 90 days across the entire cohort using mortality data and the modified Rankin Scale (mRS), with mRS ≥3 indicating poor functional recovery.

Results: Among the 125 patients, 42 (33.6%) died within 90 days, and 38 (30.4%) experienced poor functional recovery (mRS ≥3). Unadjusted analyses identified several shared clinical and radiological variables significantly associated with both mortality and poor functional recovery, respectively: advanced age >60 years (p=0.024; p=0.005), hypertension (p=0.007; p=0.045), smoking (p=0.002; p=0.040), atrial fibrillation (p=0.012; p=0.011), anemia (p=0.024; p<0.001), hematoma volume >30 cc (p=0.033; p=0.004), midline shift (p<0.001; p=0.010), and higher composite ICH scores (p=0.035; p=0.014). Furthermore, intraventricular extension (p=0.005) and severe admission blood pressure >230/140 mmHg (p=0.019) were specifically linked to increased mortality. A low admission Glasgow Coma Scale (GCS) score <8 (p<0.001) was associated with poor functional recovery among survivors. While an association was observed between neurosurgical intervention and improved functional outcomes in unadjusted analyses, this finding should be interpreted with caution, given the potential selection bias inherent in the retrospective design.

Conclusion: Warfarin-associated ICH in patients with mechanical heart valves remains a devastating complication characterized by high rates of mortality and long-term functional disability. This study identified several clinical and radiological variables associated with adverse outcomes, including advanced age, large hematoma volume, midline shift, intraventricular extension, low admission GCS, and concurrent comorbidities such as anemia and severe hypertension. Optimizing outcomes in this vulnerable population requires early risk stratification using comprehensive clinical grading systems such as the ICH score, rapid neuroimaging, rigorous blood pressure control, international normalized ratio (INR) reversal, and individualized, selective neurosurgical interventions.

## Introduction

Lifelong oral anticoagulation (OAC) is universally mandated for patients with mechanical heart valves, reducing the risk of devastating thromboembolic events by up to 75% compared to minimal or no therapy [1]. However, this vital therapy introduces a severe and competing risk: spontaneous intracerebral hemorrhage (ICH). While ICH accounts for 15-20% of all stroke cases, the absolute risk of OAC-related ICH is between 0.3 and 1% a year, compared with a spontaneous rate of 0.15% per year [2]. Warfarin-associated ICH is particularly lethal, consistently demonstrating high case fatality and severe long-term disability rates even with aggressive medical and surgical intervention. Warfarin-related ICH is associated with a mortality of approximately 50% within the first 30 days [3]. The mortality rate varies with the patient's neurological status at admission. Patients who are unconscious on arrival have a 96% mortality rate, while those who receive anticoagulant antagonists when still conscious have a mortality rate of 28% [3].

Predicting clinical outcomes and guiding acute management in this specific, high-risk population is critical but complex. Established prognostic variables - such as admission Glasgow Coma Scale (GCS) score, hematoma volume, and the presence of intraventricular hemorrhage - are well-recognized in the general population [4]. But there is limited literature on a specific cohort of patients with mechanical prosthetic valves describing their prognostic outcome and management [5]. In resource-limited settings, such as tertiary care centres serving rural and tribal populations in India, utilization of available diagnostic and prognostic tools is uniquely challenging. In these environments, delayed presentations are common, and serial neuroimaging is frequently unfeasible. Consequently, clinicians are routinely compelled to make rapid, high-stakes decisions regarding anticoagulation reversal, surgical triage, and critical care based almost exclusively on initial clinical assessments. This lack of management guidelines is compounded by the resource-limited setting (e.g., lack of serial imaging or prothrombin complex concentrate), creating an urgent need for initial-data-based prognostication. To address this specific clinical gap, this retrospective study aimed to evaluate the clinical and radiological variables associated with 90-day mortality and functional recovery in patients with mechanical heart valves presenting with warfarin-associated ICH. By identifying reliable early prognostic indicators derived from initial admission data, this study seeks to refine risk stratification and guide acute management protocols for this vulnerable population, particularly within resource-constrained healthcare settings.

## Materials and methods

Study design and setting

This was a retrospective, observational, multicenter study conducted across four government tertiary healthcare hospitals in the tribal belt of central and southern India. These centers serve as major regional referral hubs for a population of more than 1 million, primarily receiving patients transferred from smaller peripheral hospitals and rural clinics within the surrounding catchment area. Crucially, all four participating centers are equipped with dedicated intensive care units (ICUs) and have full-time on-site neurosurgical and radiological availability, allowing for the immediate multidisciplinary management of acute cerebrovascular emergencies.

Study objectives

The primary objective was to perform a clinical assessment of warfarin-induced intracerebral hemorrhage cases in patients with mechanical heart valves admitted to the tertiary medical centers in India. The secondary objective was to evaluate mortality and functional outcomes, with particular focus on the influence of risk factors and radiological findings on prognosis and disease progression.

Inclusion and exclusion criteria

The inclusion and exclusion criteria of the study are shown in Table 1. The institutional ethics committee approved the study, and all procedures adhered to institutional and national ethical standards, following Committee on Publication Ethics (COPE) guidelines [6].

**Table 1:** Inclusion and exclusion criteria for patients with intracerebral hemorrhage (ICH) of the study. ICH, intracerebral hemorrhage; CT, computed tomography; ICD-10-CM, International Classification of Diseases, 10th Revision, Clinical Modification.

Inclusion criteria	Exclusion criteria
Medical records of patients presenting between January 2015 and December 2024	Secondary causes of ICH (trauma, ruptured aneurysms, vascular malformations, or hemorrhage-prone brain tumors)
A final diagnosis of stroke (intracerebral hemorrhage), ICD-10-CM code - I61.0 to I61.9 [7]	Recent intracranial, intraspinal, or major surgeries (due to re-bleeding risk)
Presented within 24 hours of neurological symptom onset (sudden headache, loss of consciousness, aphasia, slurred speech, hemiparesis/hemiplegia, or ataxia)	Ischemic stroke or myocardial infarction within the preceding three months
Underwent non-contrast CT brain imaging on the day of admission, confirming ICH	Traumatic head injury
Documented history of mechanical prosthetic valve implantation	Presentation beyond 24 hours after symptom onset
Taking Warfarin	Patients under 18 years of age
Available follow-up records for 90 days	Pregnant women (owing to safety concerns)

Study population and patient selection

**Figure 1:**
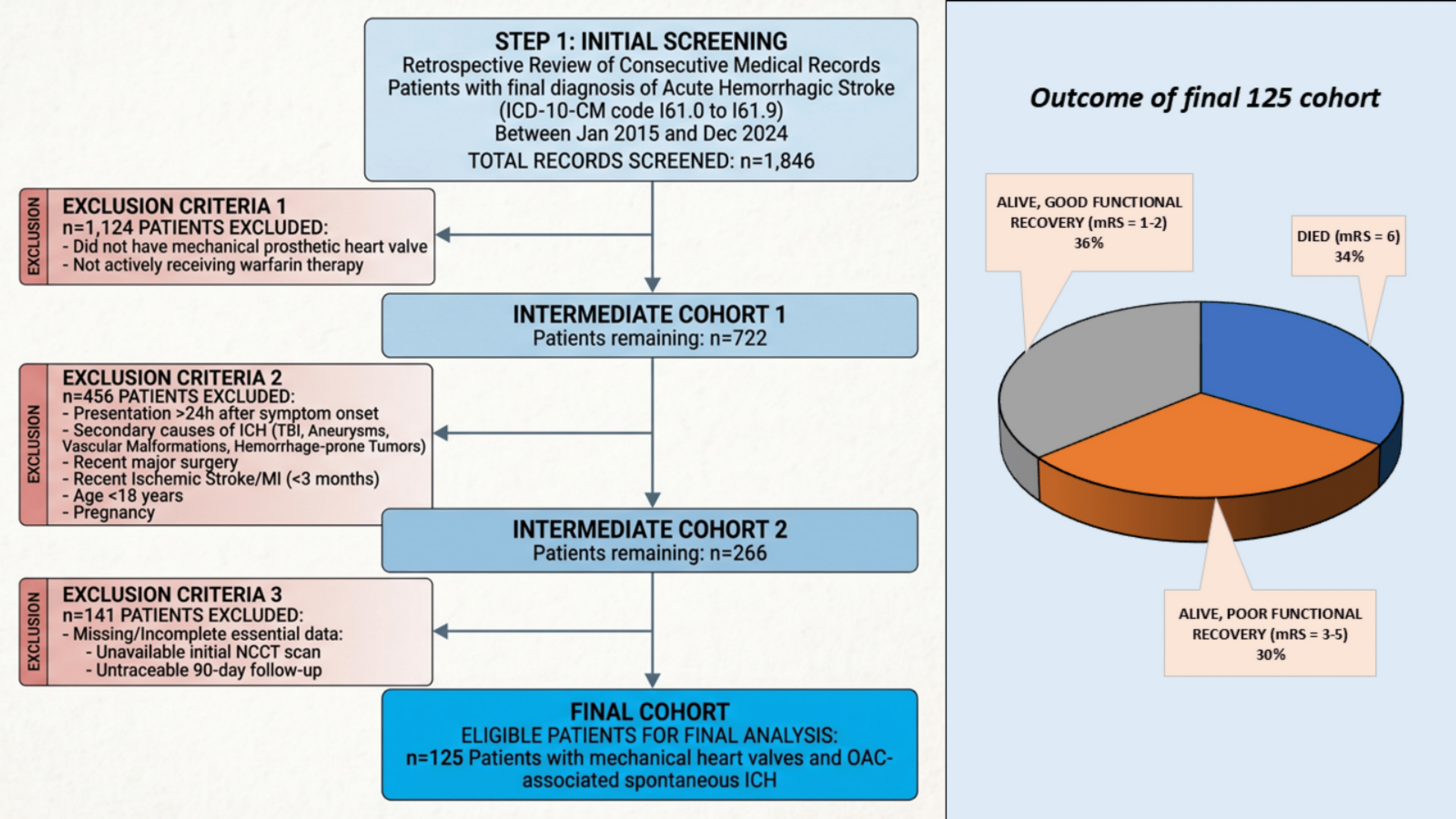
Patient selection flowchart and 90-day outcomes in the study cohort of warfarin-associated intracerebral hemorrhage (ICH) among patients with mechanical prosthetic heart valves. ICD-10-CM, International Classification of Diseases, 10th Revision, Clinical Modification; ICH, Intracerebral hemorrhage; TBI, Traumatic brain injury; MI, Myocardial infarction; NCCT, Non-contrast computed tomography; OAC, Oral anticoagulation; mRS, Modified Rankin Scale This illustration was created by the author using Microsoft PowerPoint (Microsoft Corporation, Redmond, WA, USA).

To ensure unbiased case ascertainment, we conducted a retrospective review of consecutive medical records for all patients admitted with a final diagnosis of acute hemorrhagic stroke with ICD-10-CM code I61.0 to I61.9 between January 2015 and December 2024 [7]. From 1,846 ICH records screened over 10 years, 1,124 were excluded for lacking mechanical prosthetic heart valves or active warfarin therapy; 456 more for predefined criteria (hospital presentation >24 hours post-onset, secondary ICH causes like trauma/aneurysms/malformations/tumors, recent major surgery, ischemic stroke/MI within three months, age <18, or pregnancy); and 141 for missing data (e.g., no initial CT or 90-day follow-up), yielding a final cohort of 125 patients with mechanical valves and warfarin-associated ICH (Figure 1). No significant baseline differences were found between the 125 included cases and the 141 excluded for missing data.

ICU protocols

For outpatient anticoagulation management, target INR levels were established at 2.0-3.0 for patients with isolated aortic valve replacements, and 2.5-3.5 for those with mitral or double valve replacements (per standard institutional protocols; 2020 ACC/AHA Guideline) [8]. Patients were managed in a dedicated neuro-ICU under a neurologist and critical care team. Standard protocol included systolic blood pressure (SBP) control targeting <140 mmHg, and INR reversal using vitamin K and/or fresh frozen plasma (FFP) initiated immediately following CT confirmation of ICH and documentation of an INR >5 [5]. Prothrombin complex concentrate was not available in most of the centers for most of the time. The institutional protocol consisted of 10 mg intravenous Vitamin K infused slowly over 30 minutes, administered as soon as possible or concomitantly with other reversal agents [5]. FFP was administered at a dose of 10 mL/kg. Repeat INR testing was performed 24 hours post-intervention to ensure adequate reversal. Timing of post-hemorrhage anticoagulation resumption was noted and documented across the participating centers in this study. However, the retrospective nature of the medical records restricted access to comprehensive outpatient and longitudinal data. While institutional INR targets and average time from valve surgery could be identified, the time in therapeutic range before admission (e.g., via the Rosendaal method), outpatient warfarin adherence, and interacting medications could not be reliably assessed [9].

Radiological assessment and hematoma volume estimation

**Figure 2:**
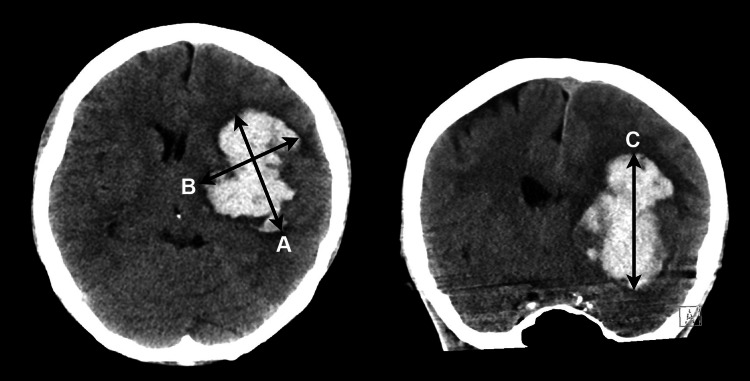
Representative computed tomography brain study image from the cohort demonstrating the ABC/2 measurement technique for hematoma volume estimation using the ellipsoid method with perpendicular diameters A, B, and C. A is the largest diameter of the hematoma on the index slice; B is the largest perpendicular diameter on the same slice; C is the sum of slice thickness contributions, assigning 1 for slices with hemorrhage ≥75% of the index‑slice area, 0.5 for 25-75%, and 0 for ≤25%. For 5 mm slices, the summed C is divided by 2. ICH volume (in cm³) is calculated as (A × B × C)/2, approximating the volume of an ellipsoid. ICH, intracerebral hemorrhage.

All patients underwent neuroimaging using 32-slice multidetector CT (MDCT) scanners with standardized protocols across the four participating centers, maintaining a constant slice thickness of 5 mm. To ensure high-quality data, hematoma volumes were independently assessed by two trained investigators (a radiologist and a neurologist) who were blinded to the patients' clinical outcomes and 90-day functional status. Inter-rater reliability was assessed, demonstrating high consistency between observers (Interclass Correlation Coefficient >0.90). Hematoma volume was calculated using the ABC/2 method, a widely accepted bedside technique with strong correlation to advanced volumetric analyses, including imaging software and three-dimensional reconstruction [10]. Although both the ABC/2 and planimetric methods are reliable, the ABC/2 method was chosen for its practicality in emergency and resource-limited settings, where rapid decision-making is essential. Each CT slice containing hemorrhage was evaluated individually; the slice with the largest hematoma area was selected for primary measurement. Dimension A denoted the greatest hematoma diameter, B the maximum perpendicular diameter, and C the sum of thicknesses across all hemorrhage-containing slices. Slices with hemorrhage <25% of the maximum area were excluded; those with 25-75% contributed half their thickness, per Kothari et al [10]. Hematoma volume (cm³) was computed as (A × B × C)/2 (Figure 2). In cases of multifocal or "satellite" hemorrhages, volumes for each bleed were measured separately and then summed to determine the total intracerebral blood burden. Intraventricular extension and hydrocephalus were documented as present or absent based on the initial non-contrast CT. While some patients received CT angiography based on clinical requirements, non-contrast CT remained the primary diagnostic tool for volume estimation across the cohort; therefore, markers of active bleeding, such as the "spot sign" and dynamic hematoma expansion, were not systematically evaluated due to the limited feasibility of routine repeat imaging in this setting.

Data collection

Patients with available follow-up records were analyzed for residual symptoms and neurological deficits. Functional outcomes were evaluated at 90 days post-ictus using the modified Rankin scale (mRS) [11]. To ensure accuracy in this retrospective setting, mRS scores were reconstructed through a comprehensive review of medical records, including neurology/neurosurgery progress notes, nursing documentation, physical therapy logs, and specific descriptions of activities of daily living. Although mRS scores were reconstructed retrospectively from clinical records rather than via prospectively structured interviews - potentially risking under-reporting of subtler cognitive or quality-of-life deficits - all outcome assessors (senior neurologists and neurosurgeons) were specifically trained in the standardized application of the mRS to minimize inter-rater variability. Retrospective recall of the 90-day mRS at a later time point is a valid means to impute missing data in stroke clinical trials, as shown by Wang et al. [12]. Outcome assessors were blinded to the initial imaging data, which provided a grounded basis for functional grading. Mortality was systematically captured for the entire cohort of 125 patients by cross-referencing institutional mortality registries and conducting structured telephone follow-ups with next of kin. For deaths occurring at external facilities, authenticity was verified by two senior consultants who reviewed digitally transmitted death certificates and discharge summaries.

Statistical analysis

Data were analyzed using IBM SPSS Statistics, version 27.0 (IBM Corp., Armonk, NY, USA), with continuous variables summarized as Mean ± Standard Deviation (SD) and categorical variables expressed as frequencies and percentages. Univariate analysis was conducted using Chi-square or Fisher’s exact tests for categorical data and Student’s t-test or ANOVA for continuous data, providing odds ratios (OR) with 95% confidence Intervals (CI) for all predictors. A p-value < 0.05 was considered statistically significant. Results were presented in structured tables and charts for clarity. Patients with missing essential data (e.g., initial CT or 90-day mRS; n=141, 7.6%) were excluded via complete case analysis, with no significant baseline differences from included cases, supporting minimal selection bias.

## Results

A total of 125 patients were included after careful review of medical records spanning the past 10 years, applying the defined inclusion and exclusion criteria. At 90-day follow-up, 42 (33.6%) had died, 45 (36.0%) were alive with good functional recovery, and 38 (30.4%) were alive with poor functional recovery(Figure 1). The baseline demographic and clinical profile of the study population is summarized in Table 2.

**Table 2:** Baseline demographic, clinical, radiological, and laboratory characteristics of the study population (n = 125). Values are presented as numbers (percentages) for categorical variables and mean ± standard deviation (SD) for continuous variables.

Variable	Number (percentage)/Mean ± SD
Age	55.2 ± 4.3 years
Sex	
Male	72 (57.6%)
Female	53 (42.4%)
Clinical Presentation	
Headache	49 (39.2%)
Vomiting	53 (42.4%)
Loss of consciousness	89 (71.2%)
Seizure	16 (12.8%)
Aphasia	23 (18.4%)
Weakness	76 (60.8%)
Ataxia	9 (7.2%)
Cranial nerve palsy	32 (25.6%)
Risk factors	
Diabetes	31 (24.8%)
Hypertension	65 (52.0%)
Dyslipidemia	33 (26.4%)
Smoking	35 (28.0%)
Atrial fibrillation	76 (60.8%)
Alcohol intake	40 (33.0%)
Mechanical prosthetic valve	
Mitral valve	52 (41.6%)
Aortic valve	38 (30.4%)
Dual valve	35 (28.0%)
Serum creatinine	1.45 ± 0.12 mg/dl
Serum hemoglobin	10.1 ± 1.4 g/dl
International normalized ratio (INR)	5.13 ± 0.79
Site of bleed	
Lobar	63 (50.4%)
Deep	46 (36.8%)
Infratentorial	16 (12.8%)
Volume of bleed	52.8 ± 9.4 cm³
Intraventricular extension	47 (37.6%)
Midline shift (>5 mm)	36 (28.8%)
Neurosurgery	32 (25.6%)

**Figure 3:**
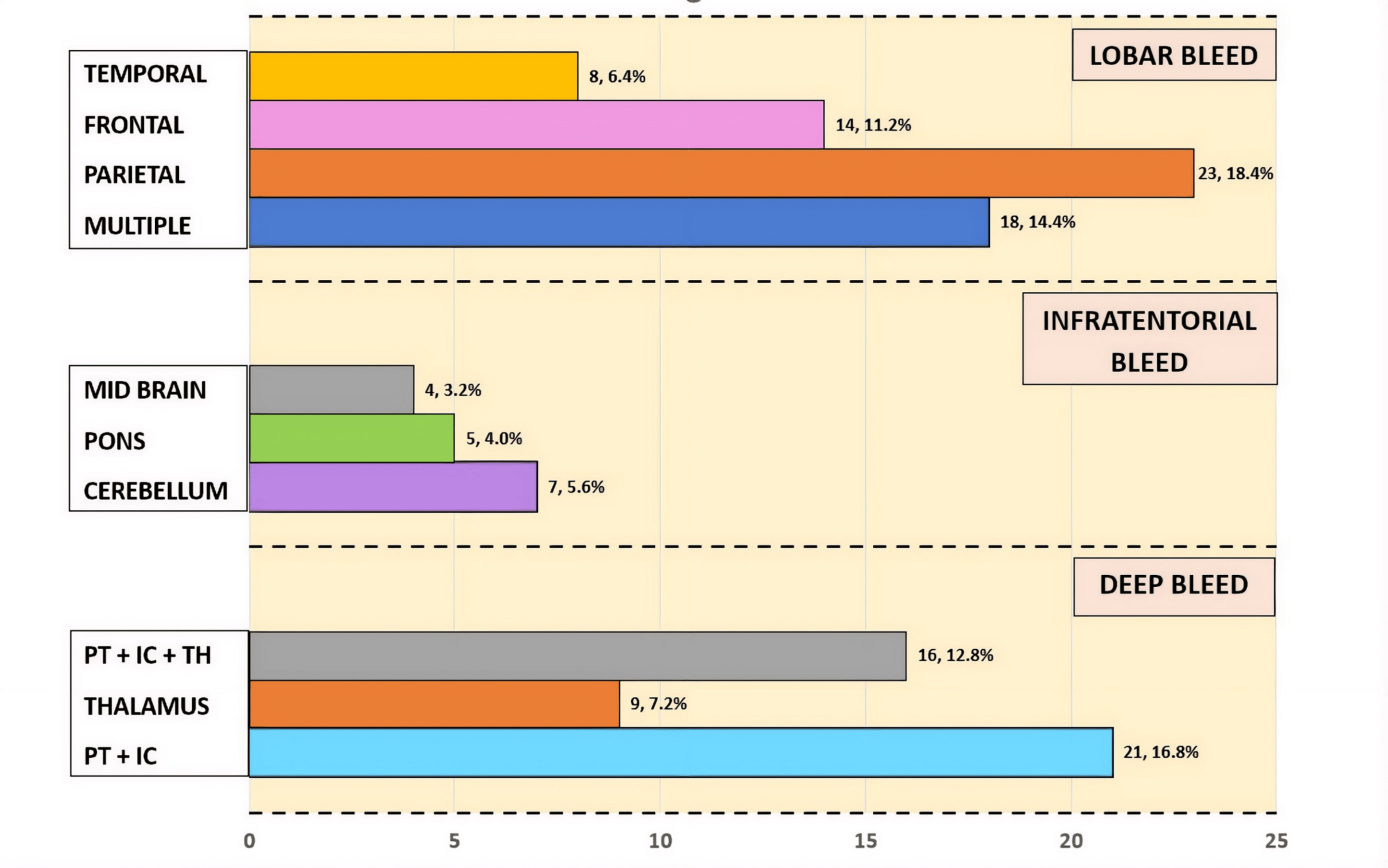
Anatomical distribution of warfarin-associated intracerebral hemorrhage in the study population. PT, putamen; IC, internal capsule, TH, thalamus.

Mean age was 55.2±4.3 years (57.6% male, n=72/125). Common presentations: loss of consciousness (LOC; 71.2%, n=89), weakness (60.8%, n=76); least: ataxia (7.2%, n=9). Risk factors: AF (60.8%, n=76), hypertension (HTN; 52%, n=65). Valves: mitral (41.6%, n=52), aortic (30.4%, n=38), dual (28%, n=35). Labs: Hb 10.1±1.4 g/dL, INR 5.13±0.79. Bleeds: lobar (50.4%, n=63; parietal 18.4% n=23>multiple 14.4% n=18>frontal 11.2% n=14>temporal 6.4% n=8), deep (putamen + internal capsule {PT+IC} 16.8% n=21 > PT + IC + thalamus {PT+IC+TH} 12.8% n=16 > TH 7.2% n=9), infratentorial (cerebellum 5.6% n=7>pons 4% n=5>midbrain 3.2% n=4); mean volume 52.8±9.4 cm³, intraventricular hemorrhage (IVH; 37.6%, n=47), midline shift >5mm (28.8%, n=36). Surgery: 25.6% (n=32) (Figure 3).

The univariate analysis identified several significant demographic and clinical variables associated with mortality, as shown in Table 3. Age >60 years (OR 3.750, 95% CI 1.227-11.463, p=0.024), hypertension (OR 2.912, p=0.007), smoking (OR 3.459, p=0.002), atrial fibrillation (OR 2.836, p=0.012), anemia (OR 2.574, p=0.024), and double valve replacement (OR 2.456, p=0.027) significantly increased mortality risk. Variables most strongly associated were midline shift >5 mm (OR 5.600, p<0.001), ICH score 4-6 (OR 2.291, p=0.035), intraventricular extension (OR 2.976, p=0.005), bleed volume >30 cc (p=0.033), and BP >230/140 mmHg (OR 3.500, p=0.019). Sex, type 2 diabetes mellitus (DM2), isolated mitral valve replacement/aortic valve replacement (MVR/AVR), and bleed location (lobar vs. deep) showed no significant association.

**Table 3:** Comparison of baseline demographic, clinical, radiological, and treatment variables between patients who died and those who survived following intracerebral hemorrhage (ICH) with mechanical prosthetic valves (n = 125). Values are expressed as the number of patients in each category. The association between each variable and in-hospital mortality was assessed using the chi-square (χ²) test, with corresponding degrees of freedom (df), p values, and odds ratios (OR) with 95% confidence intervals (CI), and the reference category for OR is indicated as “Reference”. AF, atrial fibrillation; AVR, aortic valve replacement; BP, blood pressure; DM2, type 2 diabetes mellitus; DVR, double valve replacement; GCS, Glasgow Coma Scale; HTN, hypertension; ICH, intracerebral hemorrhage; INR, international normalized ratio; IVE, intraventricular extension; MVR, mitral valve replacement.

Variable	Category	Dead (N=42)	Alive (N=83)	Χ^2^ (df)	P-value	Odds ratios with CI
Age	<45 yrs	12	30	7.438 (2)	0.024	Reference
	45-60 yrs	18	45			1.000 (0.421-2.373)
	>60 yrs	12	8			3.750 (1.227-11.463)
Sex	Male	25	47	0.096 (1)	0.757	1.126 (0.530-2.393)
	Female	17	36			Reference
HTN	Present	29	36	7.365 (1)	0.007	2.912 (1.328-6.386)
	Absent	13	47			Reference
DM2	Present	12	19	0.482 (1)	0.487	1.347 (0.580-3.130)
	Absent	30	64			Reference
Smoking	Present	19	16	9.323 (1)	0.002	3.459 (1.529-7.827)
	Absent	23	67			Reference
Alcohol	Present	15	25	0.401 (1)	0.527	1.289 (0.587-2.829)
	Absent	27	58			Reference
BP (mmHg)	<180/100	9	28	7.948 (2)	0.019	Reference
	180-230/100-140	15	39			1.197 (0.459-3.120)
	>230/140	18	16			3.500 (1.276-9.598)
MVR	Present	18	34	0.041 (1)	0.839	1.081 (0.510-2.292)
	Absent	24	49			Reference
AVR	Present	11	27	0.530 (1)	0.467	0.736 (0.322-1.683)
	Absent	31	56			Reference
DVR	Present	17	18	4.884 (1)	0.027	2.456 (1.095-5.506)
	Absent	25	65			Reference
Anemia	Present	32	46	5.127 (1)	0.024	2.574 (1.121-5.912)
	Absent	10	37			Reference
INR	>3.5	26	38	2.901 (1)	0.089	1.924 (0.902-4.106)
	≤3.5	16	45			Reference
AF	Present	32	44	6.286 (1)	0.012	2.836 (1.236-6.509)
	Absent	10	39			Reference
Antiplatelet use	Present	19	21	5.82 (1)	0.016	2.441 (1.121-5.381)
	Absent	23	62			Reference
Site (side)	Left	16	34	1.940 (3)	0.585	Reference
	Right	13	29			0.953 (0.394-2.305)
	Bilateral	9	17			1.125 (0.413-3.067)
	Midline	4	3			2.833 (0.566-14.183)
Volume of bleed	<30 cc	12	44	6.847 (2)	0.033	Reference
	30-60 cc	15	18			3.056 (1.198-7.795)
	>60 cc	15	21			2.619 (1.044-6.572)
Midline shift	<3 mm	12	48	14.931 (2)	<0.001	Reference
	3-5 mm	9	20			1.800 (0.656-4.940)
	>5 mm	21	15			5.600 (2.240-13.997)
IVE	Present	23	24	7.940 (1)	0.005	2.976 (1.377-6.433)
	Absent	19	59			Reference
Site (location)	Lobar	20	43	1.258 (2)	0.533	Reference
	Deep	18	28			1.382 (0.624-3.061)
	Infratentorial	4	12			0.717 (0.205-2.501)
GCS	<8	29	35	8.951 (1)	0.003	3.059 (1.44-6.55)
	≥8	13	48			Reference
ICH score	1-3	23	61	4.439 (1)	0.035	Reference
	4-6	19	22			2.291 (1.051–4.992)
Neurosurgery	Present	11	21	0.012 (1)	0.914	1.048 (0.449-2.445)
	Absent	31	62			Reference

Analysis of 83 surviving patients identified significant variables associated with poor functional recovery, including age >60 (OR=9.857, p=0.005), anemia (OR=5.307, p<0.001), atrial fibrillation (OR=3.250, p=0.011), smoking (OR=3.259, p=0.04), hypertension (OR=2.471, p=0.045), and neurosurgery (OR=0.28, p=0.022). Severity markers showed GCS <8 (OR=6.731, p<0.001), ICH score 4-6 (OR=3.541, p=0.014), bleed volume >60 cc (OR=5.962, p=0.004), and midline shift >5 mm (OR=7.294, p=0.01) (Table 4).

**Table 4:** Comparison of demographic, clinical, radiological, and treatment-related variables between patients with poor recovery and good recovery following intracerebral hemorrhage (ICH) associated with mechanical prosthetic valves (n = 83 survivors). Values are expressed as the number of patients in each category. The association between each variable and functional outcome at discharge was assessed using the Chi-square (χ²) test with corresponding degrees of freedom (df), p values, and odds ratios (OR) with 95% confidence intervals (CI), and the reference category for OR is indicated as “Reference”. AF, atrial fibrillation; AVR, aortic valve replacement; BP, blood pressure; DM2, type 2 diabetes mellitus; DVR, double valve replacement; GCS, Glasgow Coma Scale; HTN, hypertension; ICH, intracerebral hemorrhage; INR, international normalized ratio; IVE, intraventricular extension; MVR, mitral valve replacement.

Variable	Category	Poor recovery (N=38)	Good recovery (N=45)	Χ² (DF)	P-value	OR (95% CI)
Age	<45 yrs	7	23	10.574 (2)	0.005	Reference
	45-60 yrs	25	20			4.107 (1.466-11.508)
	>60 yrs	6	2			9.857 (1.613-60.245)
Sex	Male	20	27	0.455 (1)	0.5	0.741 (0.310-1.772)
	Female	18	18			Reference
HTN	Present	21	15	4.034 (1)	0.045	2.471 (1.014-6.019)
	Absent	17	30			Reference
DM2	Present	7	12	0.794 (1)	0.373	0.621 (0.217-1.780)
	Absent	31	33			Reference
Smoking	Present	11	5	4.212 (1)	0.04	3.259 (1.017-10.443)
	Absent	27	40			Reference
Alcohol	Present	10	15	0.482 (1)	0.488	0.714 (0.276-1.850)
	Absent	28	30			Reference
MVR	Present	18	16	1.189 (1)	0.276	1.631 (0.675-3.941)
	Not present	20	29			Reference
AVR	Present	15	12	1.540 (1)	0.215	1.793 (0.710-4.533)
	Not present	23	33			Reference
DVR	Present	11	7	2.176 (1)	0.14	2.212 (0.760-6.437)
	Not present	27	38			Reference
Anemia	Present	29	17	12.385 (1)	<0.001	5.307 (2.031-13.867)
	Absent	9	28			Reference
INR	>3.5	21	17	2.538 (1)	0.111	2.035 (0.845-4.899)
	≤3.5	17	28			Reference
AF	Present	26	18	6.681 (1)	0.011	3.250 (1.311-8.054)
	Absent	12	27			Reference
Site (side)	Right	15	14	1.375 (3)	0.711	Reference
	Left	14	20			0.653 (0.241-1.773)
	Bilateral	7	10			0.653 (0.195-2.190)
	Midline	2	1			1.867 (0.152-22.936)
Volume	<30 cc	13	31	10.930 (2)	0.004	Reference
	30-60 cc	10	8			2.981 (0.960-9.256)
	>60 cc	15	6			5.962 (1.893-18.773)
Midline shift	<3 mm	17	31	9.158 (2)	0.01	Reference
	3-5 mm	9	11			1.492 (0.516-4.311)
	>5 mm	12	3			7.294 (1.805-29.479)
IVE	Present	14	10	2.142 (1)	0.143	2.042 (0.779-5.351)
	Absent	24	35			Reference
Site (location)	Lobar	17	26	4.900 (2)	0.086	Reference
	Deep	12	16			1.147 (0.436-3.015)
	Infratentorial	9	3			4.588 (1.084-19.416)
BP (mmHg)	<180/100	2	5	1.555 (2)	0.46	Reference
	180-230/100-140	24	30			2.000 (0.356-11.230)
	>230/140	12	10			3.000 (0.475-18.930)
GCS	<8	25	10	16.036 (1)	<0.001	6.731 (2.549-17.775)
	≥8	13	35			Reference
ICH score	1-3	29	39	6.050 (1)	0.014	Reference
	4-6	9	6			3.541 (1.256–9.975)
Neurosurgery	Present	5	16	5.241 (1)	0.022	OR 0.28 (0.091-0.831)
	Absent	33	29			Reference

Poor recovery patients were older (56.8±3.9 vs 53.9±4.2 years, p=0.0017), had larger bleeds (55.9±9.8 vs 50.2±8.2 cc, p=0.005), higher SBP (208.3±32.4 vs 188.6±23.9 mmHg, p=0.0021), elevated INR (5.41±0.7 vs 4.92±0.8, p=0.0031), lower GCS (7.5±1.5 vs 9.0±2.0, p=0.0003), and higher ICH Score (4.0±1.0 vs 3.0±0.5, p<0.0001); Hb and DBP showed no differences (p>0.05) (Table 5).

**Table 5:** Comparison of clinical, laboratory, radiological, and outcome measures between patients with poor and good functional outcome following intracerebral hemorrhage (ICH) in patients with mechanical prosthetic valves (n = 83 survivors). Continuous variables are presented as mean ± standard deviation (SD) and were compared using the student’s t test and a p-value <0.05 was considered statistically significant. SBP, systolic blood pressure; DBP, diastolic blood pressure; GCS, Glasgow Coma Scale; ICH, intracerebral hemorrhage; INR, international normalized ratio.

Variable	Overall Mean ± SD	Poor functional outcome (N=38)	Good functional outcome (N=45)	T-value	P-value
Age	55.2 ± 4.3	56.8 ± 3.9	53.9 ± 4.2	3.238	0.0017
Hemoglobin	10.1 ± 1.4	9.9 ± 1.1	10.3 ± 1.6	-1.302	0.1965
INR	5.13 ± 0.79	5.41 ± 0.7	4.92 ± 0.8	3.036	0.0031
Volume	52.8 ± 9.4	55.9 ± 9.8	50.2 ± 8.2	2.885	0.005
SBP	197.6 ± 29.6	208.3 ± 32.4	188.6 ± 23.9	3.182	0.0021
DBP	132.6 ± 18.9	136.3 ± 19.3	129.5 ± 18.1	1.654	0.1019
GCS	8.3 ± 1.9	7.5 ± 1.5	9.0 ± 2.0	-3.806	0.0003
ICH score	3.5 ± 0.9	4.0 ± 1.0	3.0 ± 0.5	5.896	< 0.0001

## Discussion

This retrospective study evaluated clinical and radiological variables associated with outcome in patients with mechanical prosthetic heart valves who developed ICH while on warfarin at tertiary care centers in India. Among 125 enrolled patients, 42 (33.6%) died within 90 days, and 38 (30.4%) experienced poor functional recovery (mRS ≥ 3). These findings underscore the grave prognosis of warfarin-related ICH and are consistent with contemporary series reporting high case fatality rates and substantial long-term morbidity in this population [13].

Demographic, risk, and clinical factors

Age was strongly associated with both mortality (p=0.024) and poor functional recovery (p=0.005). Literature suggests that advanced age may be associated with reduced structural resilience and increased microvascular fragility, which can exacerbate bleeding progression [14]. This pattern aligns with the recent Intensive Care Bundle with Blood Pressure Reduction in Acute Cerebral Haemorrhage Trial (INTERACT) 3 study linking advanced age to larger baseline hematoma and poorer clinical outcomes in anticoagulant-ICH [14]. No outcome differences by sex were observed (p=0.757 for mortality; p=0.500 for recovery), consistent with modern pooled analyses and sex-stratified ICH cohorts [15]. While the higher male representation (57.6%) may reflect regional healthcare-seeking patterns, clinical outcomes appear to depend more on neurological and radiological severity than biological sex. Work by Foschi et al. similarly shows that, after adjustment for comorbidities and access, sex does not independently predict mortality or functional recovery in ICH, including anticoagulated patients [15].

Chronic hypertension was common among those who died (69.0%, n=29) and was significantly associated with mortality (p=0.007). Persistent hypertension is known to stiffen cerebral microvessels, which may predispose them to rupture even within therapeutic anticoagulation ranges. Effective blood pressure control is a critical management strategy to limit hematoma expansion [15]. This reaffirms hypertension as a major modifiable risk factor for ICH. Diabetes did not significantly affect mortality or recovery (p=0.487), in contrast to Zheng et al., who reported that hyperglycemia predicts unfavorable functional outcome [16]. Although diabetes promotes vascular disease, its limited impact here may reflect the small sample size and the stronger influence of acute variables such as hematoma volume, blood pressure, and GCS. Notably, smoking (p=0.002) and atrial fibrillation (p=0.012) were significantly associated with increased mortality in this dataset. These factors likely contribute to the underlying vascular burden and complexity of the clinical course.

Markedly elevated blood pressure (>230/140 mmHg) at presentation was significantly associated with mortality (p=0.019). Prior studies suggest that severe hypertension promotes vessel rupture and sustains active bleeding, accelerating hematoma expansion, particularly in patients with impaired coagulation [14]. This underpins the urgent blood‑pressure‑lowering protocols, which aim to limit secondary hematoma growth. Anemia (p=0.024) was significantly associated with mortality in our study. While supratherapeutic INR (>3.5) trended toward higher mortality, it did not reach statistical significance in this cohort. Anemia impairs tissue oxygen delivery, worsening ischemic and pressure‑related injury, whereas high INR indicates excessive anticoagulation, which not only triggers ICH but also perpetuates hemorrhage. Current guidelines emphasize prompt INR normalization and correction of comorbidities like anemia to reduce further bleeding and support neurological recovery [5]. Single mitral or aortic valve replacement did not significantly affect outcomes, but double valve replacement (DVR) was associated with higher mortality. DVR patients often require more intensive anticoagulation and have more complex comorbidities, increasing both bleeding risk and event severity. Low GCS (<8) was a strong predictor of mortality (p=0.003). This aligns with contemporary cohorts that rank admission GCS as one of the most useful markers for outcome triage [17]. Concurrent antiplatelet use was significantly associated with increased mortality. This corroborates recent data by Evcili et al., showing that dual therapy amplifies intracerebral bleeding risk and mortality and underscores the need for further evaluation of CAD and careful risk-benefit assessment of concurrent antiplatelet therapy. in patients with mechanical heart valves [18]. The mean duration of restarting warfarin in our cohort was 12.5 ± 1.2 days. Literature on anticoagulation resumption after ICH in mechanical heart valve patients remains markedly underdeveloped, lacking landmark RCTs and relying on retrospective data. Kuramatsu et al. first studied 137 mechanical heart valve-ICH patients, finding restarting warfarin within two weeks increased bleeding despite thromboembolism prevention, highlighting the evidence void [19]. AHA/ASA 2022 ICH guidelines offer only Class IIb recommendations (consider restarting seven to eight weeks post-ICH), explicitly noting no Class I evidence for MHV-specific timing [20].

Radiological factors

In this study, anatomical location (lobar, deep, infratentorial) (p=0.533) and side of hemorrhage (p=0.585) were not significantly associated with mortality. Although lobar hemorrhages were frequent (50.4%), topography alone did not dictate survival in this anticoagulated cohort. This differs from some series suggesting topography influences risk and may reflect the overriding impact of total blood burden in warfarin-associated ICH [21]. With respect to side, left‑ and right‑sided bleeds showed similar mortality (32.0% and 30.9%, respectively), whereas midline hemorrhages, though uncommon, carried substantially higher risk (57%) of mortality. This is consistent with prior observations that midline or brainstem ICH, frequently involving deep neural or cardiorespiratory pathways, leads to rapid deterioration and limited therapeutic options [17]. Larger hematoma volume - particularly >30 cc - was a strong predictor of death (p=0.033). Large volumes can raise intracranial pressure, promote herniation, and compress perilesional structures. In anticoagulated patients, uncontrolled bleeding accelerates volume gain, often overwhelming compensatory mechanisms. Data from the recent Early MiNimally-invasive Removal of IntraCerebral Hemorrhage (ENRICH) trial confirm that outcomes worsen sharply as size increases, with hematomas exceeding 60 cc frequently associated with poor outcomes or death [22]. Midline shift >5 mm and intraventricular extension (IVE) were significant imaging variables associated with mortality (p<0.001 and p=0.005, respectively). Midline shift indicates significant mass effect and impending herniation, while IVE increases the risk of acute hydrocephalus and secondary inflammation, worsening the prognosis in anticoagulated patients.

Neurosurgical factors

The role of neurosurgery in this cohort requires careful interpretation. Neurosurgical intervention was not significantly associated with a reduction in 90-day mortality (p=0.914). However, it was associated with improved functional recovery among survivors (p=0.022; OR 0.28). These findings suggest that while surgery may not have prevented death in the most severe cases, it was linked to better outcomes in selected patients. However, this association must be viewed with caution due to the selection bias inherent in a retrospective design, where patients with extremely poor prognoses may not have been offered surgery. The decision for surgical intervention in our setting was likely individualized based on hematoma volume and neurological status, consistent with guidelines recommending tailored management based on clinical severity and mass effect [23]. Existing literature remains divided; while some trials suggest minimally invasive methods may reduce mortality, the evidence is often neutral. While the ENRICH trial demonstrated improved functional outcomes with early minimally invasive surgery in selected ICH patients, the landmark STICH I and II trials showed no overall functional benefit from early craniotomy versus medical management for supratentorial ICH, highlighting the importance of surgical technique and patient selection in this cohort [24]. There is no separate literature on patients with mechanical heart valves and ICH.

Follow-up functional outcomes

A higher composite ICH score, which integrates age, GCS, volume, IVE, and location, robustly associated with death (p<0.001) and poor functional recovery (p=0.011). Composite scoring outperforms single variables in capturing the complexity of stroke outcomes. Multiple external validation studies, including large Asian (Hegde et al.) and Western ICH registries (Aysenne et al.), have confirmed its accuracy, making it a cornerstone for triage and decision-making in acute ICH [24,25]. In the present study, functional outcomes were assessed using the mRS, a globally accepted measure for evaluating post-stroke recovery and disability [11]. The mRS categorizes patient outcomes from 0 (no symptoms) to 6 (death), with scores of 0-2 commonly accepted as indicating functional independence and scores ≥3 as indicating moderate to severe disability or dependence. Among the 83 survivors, only 45 achieved a good functional outcome, while 38 remained functionally dependent. Patients with good outcomes typically presented with smaller hematoma volumes (<30 cc), higher GCS scores on admission (≥8), no midline shift, therapeutic INR levels, no anemia, and often have a lower ICH score. For instance, a 45-year-old with a 24-cc hematoma, GCS 13, and INR 3.9 achieved full independence (mRS 1), while a 67-year-old with a 58-cc lobar hemorrhage, INR 9.6, and midline shift remained bedbound (mRS 5) despite intensive care. These outcomes highlight the persistent morbidity associated with warfarin-associated ICH. Multiple contemporary studies support the conclusion that acute modifiable factors-such as hematoma size, neurological status (GCS), and mass effect (midline shift)-play a more defining role in outcomes than certain baseline demographics [26]. Landmark mechanical valve ICH anticoagulation literature remains underdeveloped; the 2022 AHA/ASA ICH guidelines recommend that patient selection for surgical evacuation, INR reversal, and intensive blood pressure management be tailored based on clinical severity and imaging features such as midline shift and hematoma volume, all of which are key determinants of mRS outcomes [20]. In a systematic review by Kuramatsu et al., warfarin-associated ICH in patients with mechanical heart valves was particularly associated with worse outcomes when initial treatment failed to address these acute risk modifiers [19]. Optimizing outcomes requires early risk stratification using the ICH score, rapid neuroimaging, and aggressive management of acute risk modifiers.

Limitations

This study has several important limitations. We regret that its retrospective, observational nature and the use of unadjusted univariate analyses allow for identifying associations only, rather than establishing causal "predictors" or "protective" effects. The absence of multivariable modelling, multiplicity control for numerous comparisons, and sensitivity analyses requires these findings to be interpreted as exploratory clinical associations within a resource-limited cohort rather than a validated prognostic model. Data regarding time in therapeutic range, outpatient warfarin adherence, interacting medications, and specific heparin bridging protocols could not be reliably captured from the available medical records. Our radiological analysis was restricted to initial admission data; due to resource constraints and the clinical priority of immediate management, markers of active bleeding (such as the CT "spot sign"), quantitative intraventricular blood volume, and dynamic hematoma expansion via repeat imaging were not systematically evaluated. Finally, the manual extraction of variables from disparate electronic and paper records across four centers introduces the potential for minor inaccuracies, notwithstanding a secondary audit performed to ensure internal consistency across the regenerated results.

## Conclusions

In conclusion, warfarin‑associated ICH in patients with mechanical heart valves is a devastating complication, with about one‑third of patients dying and another third left with significant 90‑day disability. Adverse outcomes are linked to advanced age, low admission Glasgow Coma Scale, anemia, severe hypertension, large hematoma volume, substantial midline shift, and intraventricular extension. Strict anticoagulation monitoring and cautious use of dual antithrombotic therapy are essential. These findings support early risk stratification with the ICH score and guideline‑directed management focused on rapid neuroimaging, strict blood pressure control, and prompt reversal of supratherapeutic anticoagulation. The observed association between neurosurgery and outcome should be interpreted cautiously because of probable selection bias, reinforcing the need for individualized surgical decisions. Overall, this study underscores the urgency of rapid, protocolized care in resource‑limited tertiary centres and the need for prospective, multivariable studies to better define prognostic factors and optimize treatment strategies in this high‑risk group.
